# Beitrage zur Physiologischen und Pathologischen Chemie und Mikroskopie, in ihrer Anwendung auf die praktische Medizin unter Mitwirkung, &c.

**Published:** 1843-07-01

**Authors:** 


Beitrage zur Physiologischen und Pathologischen Chemie und Mikroskopie, in ihrer Anwendung auf die praktische Medizin unter Mitwirkung, &c. Herausgegeben von Dr. *F. Simon*. Band 1. Lieferung 1. Bogen 1—9. Berlin, 1843.

This is a new periodical, to be devoted exclusively to Chemistry and Microscopy, in their applications to Physiology and Pathology. The Editor is already favourably known to the public by his work on Medical Chemistry, as also by numerous contributions to pathological chemistry. It is our intention to extract from his Journal whatever may appear to us to possess sufficient interest for British readers. The introductory article, the professed object of which is to show *what Chemistry has done for Practical* Medicine, we have condensed on the present occasion. It contains the researches of the most eminent chemists into the composition of the principal fluids of the body, and the various changes and modifications produced in them by disease, and shews how far such modifications may be rendered available in practical medicine in establishing the Diagnosis, Prognosis, and Treatment of Disease.

THE BLOOD.

The physical characters of the blood are known to us ever since Leuenhoek's time; its chemical composition was not investigated till a later period. Dumas and Prevost made the first accurate chemical enquiries, especially into the constitution of the blood. Lecanu succeeded in isolating the colouring matter of the blood first in combination with protein, and afterwards perfectly. Denis instituted numerous experiments on the chemical constitution of the blood, more especially with respect to the relative proportions in which the corpuscles of the blood exist therein, as compared with the fibrine, albumen, the salts, and the fat. Müller has shewn that the fibrine, albumen and globuline contained in the blood, consist chiefly of protein, and accordingly must be considered as slight modifications of one and the same organic substance; more recently still, Andral, Gavarret, and Simon have studied the different constitution of the blood in diseases; these chemical enquiries, combined with a correct appreciation of the physical state of the blood under various circumstances, have become important for diagnosis, and even for prognosis and treatment. Already the old physicians know how to deduce from the coagulation of the blood, from the quality of the coagulum and of the serum, conclusions respecting the process of disease, which they attained through experience, and the correctness of which was confirmed by continued attentive observations; the latest enquiries have taught us to become acquainted with those qualitative changes, and have shewn their connexion with the different chemical conditions of the blood, and this knowledge of the different chemical conditions of the blood has yielded the most important contributions to semeiology through the medium of this fluid.

From *the manner in which the coagulation of the blood takes place*, when it is slow, but yet complete, in which case the blood has a higher temperature than in the healthy state, we may infer the existence of a phlogistic state; from an imperfect coagulation, when it does not amount to the formation of a perfect coagulum, but at most to the separation of gelatinous flocculi, a deficiency in fibrine is to be inferred. This then again indicates a high degree of debility and a depression of the vitality of the blood; this state of the blood is characterized as dissolution, though in most cases those parts which in the living blood are not dissolved, but are only floating, viz. the corpuscles of the blood, are observed to be in a state of perfect integrity.

From the *quality of the coagulum* we infer, when it is large and firm, a great quantity of fibrine, and at the same time a phlogistic state, more especially if the serum is clear and of a bright yellow, and the coagulum is covered with a solid inflammatory crust. But from a large solid coagulum we are not warranted in inferring a large quantity of blood-corpuscles, as in the generality of cases a diminution of the blood-corpuscles goes hand-in-hand with the increase of fibrine; accordingly a large solid body of blood, exclusive of the blood-corpuscles, must include a no inconsiderable quantity of blood-serum, for then the inflammatory crust itself is but a blood-serum inclosed in coagulated fibrine. A small solid coagulum indicates deficiency in the blood-corpuscles, there existing at the same time diminution, or at all events no increase of the fibrine, a state which is observed in chlorosis. A soft, dissolved, bilious-like coagulum indicates great deficiency in fibrine, as observed when the vital powers are depressed, as, for instance, in the far advanced stage of typhus.

When the serum is very dark-coloured, we may infer that the red-colour of the blood is dissolved, or that the colouring matter of the bile is present; the former is ordinarily the case, when the blood contains an excess of water, or an excess or deficiency in the salts; in such cases also the quantity of the fibrine is extraordinarily diminished, and it belongs only to an imperfect formation of the coagulum; such a quality of the blood is observed in hydraemia, scurvy, and some species of bad typhus; the serum may be coloured blood-red by the colouring matter of the bile, and then, when decomposed with nitric acid, it yields the known changes of colour. In cases where the colouring matter of the bile is present in the serum, a large firm coagulum may form with an intensely coloured inflammatory crust, which indicates an inflammatory state, or the blood may coagulate only imperfectly, as in typhus icterodes. The presence of the colouring matter always indicates that the hepatic system sympathises. A whitish, turbid, milk-like serum is observed, when fat, pus, or fibrin, precipitated in very small kernels, visible only with the microscope, is made to float therein. The microscope shews which of these substances is present in the serum; if it be fat, the fat globules are seen; if pus, the pus-corpuscles; if fibrine, numberless small globules or points, which do not disappear on the addition of æther, nor on the addition of acetic acid, are deposited from the serum when diluted with water, and may be washed away with water. The presence of fat in the serum indicates not infrequently an organic change in the chylopoietic system, chiefly in the liver, as scirrhus of the liver. Pus in the serum indicates suppuration in the blood-vessels, phlebitis, or the presence of pus in the large organs, which assist in the circulation or preparation of the blood; at the very most minutely divided fibrine floating in the serum has been observed, and that but seldom. I found it but once in the blood of a man who laboured under Bright's disease of the kidney.

It is also of importance to take into our observations the form of the blood-corpuscles also; to be sure this seems to be essentially changed in but very few cases; several observers however agree in this, that in cases of severe typhus, where ammonia forms in the blood, the blood-corpuscles become changed, so that their edges appear torn and tattered.

The chemical investigation of the blood affords the most important points for establishing the diagnosis; it is evident that the correct appreciation of the signs, hitherto adduced from the physical qualities of the blood, was first obtained by physical examination; it gives us the only true solution regarding the quality and the change in the composition of this fluid; it has taught us that in inflammations the fibrine and fat become increased, and the blood-corpuscles diminished; that in chlorosis the blood-corpuscles are sometimes extraordinarily diminished, but the fibrine usually appears in its normal quantity; that in typhus the imperfect coagulation of the blood is to be ascribed to a deficiency in fibrine-matter; that in sea-scurvy an excess of salts is present; the chemical examination it is, which brings those physical signs from the blood already known to the ancients and correctly appreciated in reference to the mode of treatment into a peculiarly clear light. Experience and chemical examination have taught us, that in case of an increased reaction between the blood and oxygen the fibrine becomes increased and the blood-corpuscles diminished, and that in case of impeded re-action the quantity of fibrine becomes diminished. The results of experience also seem to lead us to think that a blood rich in fibrine increases the impulse of the heart, whereby the circulation becomes accelerated; when by abstracting blood the absolute quantity of fibrine is diminished, it appears that by this process also an impression is made on the heart's impulse, the consequence of which again is a smaller fibrination of the blood. Excessive venæsections may accordingly render the blood poor in fibrine, and thereby so change the re-action of the vascular system, that it becomes what is commonly designated a nervous re-action. From the blood and the re-action accordingly the physician must derive his indication, whether the venæsection is to be continued or not; if diminished re-action between the blood and oxygen diminishes the quantity of fibrine, it gives reason to think, that when phlogistic states exist under certain conditions, a reaction and a quality of blood may be found, which is more indicative of a nervous than of an inflammatory state. In case of inflammation of the respiratory organs, or of those inflammations which take on an extremely rapid and intense course, the physician sometimes finds a small oppressed pulse, which by itself alone, exclusive of the connexion with the other phenomena, would by no means call for venæsection; the blood drawn forms a soft, diffluent coagulum sometimes covered with a bilious-looking film, and it is only when, after a correct appreciation of the morbid process, the venesections have been repeated, that the character of the inflammation shews itself in a manner not to be mistaken, as well in the quality of the blood as in the re-action. There is not a doubt that here, by excessive congestion in the lungs, or in the entire capillary system, the reciprocal action between oxygen and the blood was diminished, and it was only after the circulation again became free by the necessary abstraction of blood, those peculiar changes produced by inflammation showed themselves as well in the blood as in the re-action. With the other phenomena, which enable the physician to recognise chlorosis in the diseased body, it is the changed composition of the blood, ascertained by chemical examination, which is expressed in the great diminution of the blood-corpuscles; this examination will also teach him how far the preparations of iron must be continued, so that by the action of these remedies the composition of the blood may be again brought back to the normal state.

### THE URINE.

*From the physical and chemical state of the urine* the attentive observing physician might obtain a great quantity of information for ascertaining and establishing a diagnosis; much of what might be said here is already known, and I shall touch on these points very superficially, much more however might prove to the reader not at all uninteresting.

The old physicians considered the examination of the urine as an important point for judging of diseases and of their probable course, and as we have already remarked, they made up for their deficiency in chemical knowledge by sharp and close observation: the earliest chemical examinations of the urine occurred in one of the earliest epochs of organic chemistry. Among the earlier investigators who paid attention to the urine, though very partially, I may mention Brandt, Kunkel, Boyle, Bellini; Boerhaave, however, attempted an analysis of the urine which, considering the time, was extremely good. Scheele's discovery of uric acid, and Cruikshanks' of urea, contributed essentially to a more correct knowledge of this secretion. The latter surgeon had already examined the urine in several diseases, especially in diabetes and dropsies. At the commencement of the present century it was chiefly Berzelius and Prout who made the urine the subject of extended enquiries; Berzelius demonstrated the existence of lactic acid, which by the earlier chemists had been considered to be acetic acid; the analysis communicated by Berzelius in 1809 of the composition of the urine, has been till within the last few years the only correct examination of the same; Prout has continued his inquiries up to the latest period. Of the more recent works on the constitution of the urine, those by Lecanu are the most prominent; within the last years Becquerel, Lehmann and Simon have employed themselves with examinations of the urine in the healthy and morbid state. Several constituents of the urine, both in the state of health and disease, are very accurately known, as uric acid, urea, lactic acid, the salts and the sugar of the urine; of others probably not less important we have a very imperfect knowledge, as of extractive and colouring matters. Regarding the quantitative composition of the urine, which is rather changeable, numerous investigations have been made by the above-named chemists. Lecanu also investigated the varieties which may be shewn in healthy urine, according to age and sex.

The quantity of urine passed in the 24 hours, and its colour, are frequently of importance. A diminished quantity of the urine passed in 24 hours is under circumstances a sign particularly of acute diseases; an excessive increase of the urine, if permanent, is oftentimes indicative of serious diseases. A dark-coloured, flaming or fiery red urine commonly indicates an inflammatory affection; a dark brown red is generally observed in typhus. But the urine may also be coloured blood-red or brown-red by bile-pigment, which is easily detected by its re-action with nitric acid; the latter constantly indicates an affection of the liver; a blood-red urine commonly contains blood; there is then for the most part found in it a sediment of blood-corpuscles, which are recognized with the microscope; but should a little blood be contained in the urine and this in a state of solution, it may be discovered by adding nitric acid, which occasions a precipitation of coagulated albumen coloured red by hæmatine. This bloody urine indicates a bleeding in the kidneys, bladder, urethra, or, in the case of women, of the uterus. Blood flowing from the urethra comes in drops. If the blood is discharged in masses after clear urine, it comes from the bladder, and in that case it often stops up the passage from the bladder by coagulation; if the blood is distributed through the urine, partly dissolved, and not in very large quantity, it comes from the kidneys; if it be dark, and mixed with mucus and pus, it owes its origin to an ulcer. The presence of stone-colic shews that the blood has been poured out during the descent of a renal calculus.

Blue urine has been observed, though not frequently; in the majority of cases it probably owes its origin to the use of certain medicines; black urine has likewise been observed; the connexion however is not yet known between the colouring matter and the morbid process; greenish urine indicates, according to Prout, an oxalic acid diathesis; sediments of oxalate of lime form, or mulberry calculi pass away; a urine, which is pale-coloured, and has a bias to green, frequently indicates the presence of albumen, which is readily detected by heating to boiling or by nitric acid. In this case the urine is not perfectly clear, but slightly opalescent; its quantity may be increased, diminished, or natural. The oxalic-acid diathesis of the urine indicates, according to Prout, functional disturbances in the chylopoietic system; albuminuria ordinarily indicates dropsy and an affection of the kidneys. The re-action of the urine is important for the physician. Natural urine, it is well known, has an acid re-action: the quantity of free acids in the urine and the intensity of the re-action may encrease to an extraordinary degree in diseases, more particularly in rheumatism, gout, in disturbances of the digestive organs, and in certain stages of typhus; to judge correctly of the intensity of the acid re-action, reference must be had to the quantity of the urine; the greater or less acid re-action is known by the effect of the urine on litmus paper of a weak blue colour, which becomes coloured so much the more rapidly and the more deeply reddened, the greater the acid contents of the urine are. Urine with a neutral re-action commonly forms the transition from the acid to the alkaline re-action, and vice versâ. The alkaline re-action of the urine is of great importance to the physician; it commonly depends on carbonate of ammonia, the presence of which is recognized by the odour, and the white cloud, which a glass-rod developes when moistened with an acid salt and brought near to it. The urine also may have an alkaline re-action through its containing carbonate of soda, which salt finds its way into the urine by the long-continued use of carbonate or bicarbonate of soda with vegetable acids. The urine alkaline by carbonate of ammonia is but seldom evacuated in this state from the bladder; during its discharge it is commonly neutral, and becomes alkaline only in a shorter or longer time after; badly-cleaned vessels may moreover contribute much to this, a circumstance which ought to be taken into account. Urine which already on voiding it has an ammoniacal re-action, and has also a very bad smell, indicates always a serious affection of the nervous system, and especially of the spinal cord. In certain unfavorable stages of tabes dorsalis, phthisis of the spinal cord, paralysis of the lower extremities and of the bladder, the voiding ammoniacal urine is ever an unfavourable sign; in other affections of the nervous system also, as in typhus, ammoniacal urine is observed, which however assumes this re-action in the majority of cases not till after it has stood for some time. In typhus the re-action of the urine may be of importance for the prognosis when the urine, after it was observed to have an acid re-action through one, two, or three periods of seven days, is finally found to be neutral, and then to have an ammoniacal odour and re-action; when this re-action lasts for several days, probably during one entire period of seven days, and then again passes into the acid, this seems in most cases to indicate a favorable termination to the disease. The urine having an ammoniacal re-action in typhus has usually a dirty, turbid yellow-brown or red-brown appearance, and forms sediments which disappear in a great measure on the addition of free acids; also in catarrhus vesicæ, or in phthisis vesicæ the urine becomes ammoniacal in a very short time after being voided; the large quantity of vesical mucus or pus indicates this affection; finally, the formation of urinary concretions, consisting of earthy phosphates, is in part occasioned by the neutral or alkaline re-action of the urine; the urine voided in this urinary affection, is not so dark as the urine in typhus, and commonly forms sediments of phosphate of lime, and of ammonio-magnesian phosphate. If the vesical calculus exercise an irritating influence on the parietes of the bladder, a great quantity of vesical mucus is commonly mixed with the urine.

The specific gravity of the urine, though by itself it possesses no great diagnostic value, as it depends on the variable quantity of water in the urine, may however claim the attention of the physician under certain circumstances; the clearer and the more like water the urine appears, the less is its specific gravity; the deeper and darker-coloured, the higher the specific gravity. This general law may admit an exception in one case, namely in diabetes mellitus; in this disease a urine is voided either normal or pale, seldom deeply coloured, the high specific gravity of which (1020—1060) is in contradiction to the colour; this high specific gravity imperatively requires a more strict examination of the urine. More than all other signs the correct examination of the sediments is of importance for the physician. Healthy urine forms only after long standing a light, sinking cloud of vesical mucus; every other separation in the urine is of a pathological nature. The urinary sediment consists either of organic formations, as mucous corpuscles, purulent corpuscles, blood, &c. or of heavy, insoluble salts or acids, or lastly of an admixture of both; the microscope will throw light on this.

The sediment consists of organic formations. If the urine has not a blood-red colour, the sediment is white, grey, dirty-yellow, and with the microscope can be seen mucus, or pus-corpuscles; here the sediment is constantly mucus, if the urine contain no albumen; it is probably pus, if the sediment is deposited rapidly after the urine is voided, and the urine contains albumen. It is not necessary now to state of what importance it is to discover and appreciate mucus and pus in the urine; in catarrhus vesicæ the mucous sediment frequently assumes a very glutinous quality; this however happens only when the urine begins to become ammoniacal, which in urine containing mucus often occurs in a very short time, as we have already mentioned; the same may be said of pus, and it is good in this case to test the presence of albumen not by boiling heat, but by nitric acid. If the sediment is blood, the blood-corpuscles are then seen with the microscope; the urine standing over this is also of a blood-red colour. Of the import of blood in the urine I have no remark to make. If the urine contain albumen, and there exist at the bottom a mucous sediment, it is of great importance to examine this with the microscope. We may find therein, as I have observed, peculiar long prominences, partly filled, partly transparent, and round spheres, twice or thrice as large as mucous-corpuscles, filled with dark, granular contents, which beyond a doubt have their origin in the kidneys, and denote a morbid state of this organ. These peculiar forms I have frequently and at different times found in the urine of a person labouring under morbus Brightii.

The sediments which are not of an organic nature, may in like manner be easily recognized with the microscope and some few re-agents; they are either crystalline or amorphous, present themselves either in acid, neutral or alkaline urine, and are readily distinguished; in acid urine sediments of uric acid present themselves, urate of ammonia, urate of soda, oxalate of lime, cystin. The greatest number of sediments which present themselves in acid urine consist of urate of ammonia; less frequent are those consisting of uric acid, still rarer are those consisting of oxalate of lime, and the most uncommon of all are those consisting of cystin. Sediments consisting of earthy phosphates do not occur in urine having a strong acid re-action. Every sediment occurring in acid urine from yellow to brown from red to purple red, appearing under the microscope as an amorphous precipitate, or as large and small globules aggregated together, which is dissolved entirely or almost entirely on warming the urine, is urate of ammonia; to this belong accordingly all the so-called critical separations in the urine; the species of separation of the urate of ammonia is very various, and it appears sometimes as mere turbidness, without forming any sediment whatever, sometimes it lies at the bottom of the vessel as coloured mucus or pus, at other times heavy, like an earthy precipitate. In the case of those diseases, which in the course of their development admit a termination by a critical separation in the urine, the kind of separation is of importance. The heavier the sediment lies at the bottom, and the clearer the urine is that stands over it, the more decided is the crisis allowed to be; whilst the lighter the sediment floats, and the less disposition there is to a perfect deposition, the more imperfect is Nature's effort to break down the disease by a crisis. The various colourings of the sediment are characteristic for some diseases; in acute rheumatism of the joints, in intermittent fevers, the critical sediment is observed to be coloured red up to a brown red; in acute diseases of the liver the sediment is rose-red; in typhus it has a dirty-red colour; in some diseases the appearance of the sediment appears to be of no constant critical import, as, for example, in typhus.

A sediment in the acid urine, which is not dissolved on heating the urine, and which appears crystalline when observed either by the unarmed eye, or with the microscope, and is coloured yellow up to vermillion red, is uric acid. It ordinarily appears in the form of rhombic plates, and in the majority of cases mixed with urates of ammonia, where it then forms the undermost and dark-coloured layer of the sediment. That the deposit of uric acid is of critical import, is scarcely to be doubted; in gout and in cases of renal calculi, where the deposits consist of uric acid, uric acid or the discharge of gravel form the most perfect crisis; in many other diseases we still want the necessary observations concerning the critical value of uric acid secretions. The sediment of oxalate of lime presents itself more rarely than those before-mentioned; it usually forms a white precipitate; observed with the microscope, it appears in the form of small octoedra, or of little spheres arranged one by the other; it is not soluble in acetic acid, but readily in hydrochloric acid; when sulphuric acid is poured on it it disappears, and after some time long, lancet-formed plates of sulphate of lime are seen.

Respecting the diagnostic value of the oxalate of lime in the sediment sufficient observations are still wanting; it is probable that it is connected with serious disturbances in the chylopoietic system; that we should be attentive to the possible formation of stone of oxalate of lime, where the sediment shews itself frequently and permanently in the urine, is of importance; however, the physician, in order to judge of the phenomena more correctly, must also have reference to the diet, as oxalic acid may be conveyed into the body by various sorts of food.

The occurrence of cystin in the urinary sediment is very rare. It is easily recognised by its remarkable form; it forms faint yellow-coloured hexaedral plates. According to Prout, the appearance of cystin in the urine is a very unfavourable sign, it indicates the formation of cystin calculi.

In neutral urine, or that with an alkaline re-action, besides the sediments already mentioned, precipitates of earthy phosphates present themselves; they are readily known by this; that they disappear on acidifying the urine with acetic acid or acid salts: the phosphate of magnesia, commonly combined with ammonia, is distinguished by its crystalline form; it appears in colourless prisms obliquely truncated, very frequently in the form of a roof; the calcareous phosphate appears almost always as an amorphous precipitate; as the earthy phosphates are constantly present in the normal urine, their precipitation is commonly to be looked on as a consequence of the formation of ammonia, the free acids by which the earthy phosphates were previously dissolved being neutralised by this alkali; in some cases, on the contrary, the appearance of earthy phosphates in the sediment is of diagnostic value. In affections of the spinal chord the phosphate of magnesia, more especially, appears to be secreted in great quantity; in affections of the mucous membrane of the bladder, the phosphate of lime appears in large quantity; in three cases of inflammations of the respiratory organs, at the time when resolution of the disease set in, I have seen the previously acid urine become neutral, and have observed, as a precipitate, the secretion of a considerable quantity of already formed crystals of ammonio-magnesian phosphate, perceptible to the naked eye, at the same time that in two of these cases the clear urine held so large a quantity of urate of ammonia in solution, that precipitates of uric acid were instantly produced by every acid. When calculi of the bladder are present, which consist of earthy phosphates, the urine frequently contains sediments of earthy phosphates, with which a greater or smaller quantity of mucus is mixed. In scarlatina the urine is observed to be turbid at the time of the desquamation, often also before the occurrence of the same on the outer cuticle; when it is observed with the microscope, an extraordinarily great quantity of the epithelium of the vesical mucous membrane is seen in it. It is, therefore, to be admitted, that the desquamation goes on also on the mucous membrane of the bladder; and if, as frequently appears to be the case, the scaling off takes place earlier on the mucous membrane of the bladder than on the external skin, one may determine the commencement of the scaling off by examining the urine.

The knowledge of the chemical composition of the urine is of very great value for diagnosis and prognosis; especially as far as concerns the presence of matters which are not found in the normal state of the urine: albumen is readily discovered in the urine by heat or by the addition of nitric acid; if the urine is acid, heat is preferred; if alkaline, nitric acid. The presence of albumen in the urine is always of great import, and the correct estimation of the same as a means of diagnosis not altogether easy: cases are known in which albumen was observed in the urine of healthy individuals, or set in in consequence of disturbance occurring in the digestive organs; in the great majority of cases albuminuria is the attendant of dropsical phenomena, or the forerunner of them, the urine is then commonly clear, evinces a tendency to green, and contains much albumen; but there are cases of dropsy known, which set altogether without albuminuria. That the presence of albumen in the urine does not infer the presence of Bright's degeneration of the kidneys, has been sufficiently proved; where degeneration of these organs is suspected, great attention must be directed to the mucous sediment of the urine. In violent inflammations, as well as in typhus, small quantities of albumen are sometimes found in the urine; the urine is then generally very dark, and has an acid re-action; according to Becquerel, this appearance of albumen in cases of inflammation appears to be connected with a congestive state of the kidney, and as this, unless the disease is itself an inflammation of the kidneys, seems to occur only in very violent and intense inflammations, the appearance of albumen in the urine accompanying inflammation might be a sign for the intensity of the inflammation. In consequence of inflammatory exanthems, especially in the desquamatory stage of scarlatina, albumen sometimes is observed to exist in the dark-coloured urine, and sometimes, though more seldom, blood. It is, therefore, a matter of importance for the physician carefully to examine the case, as this heterogeneous mixture is not infrequently the fore-runner of dropsy; however, dropsy has been observed after scarlatina without albumen in the urine, and albumen in the urine without dropsy following thereon. At the commencement of diabetes mellitus, albuminuria is no rare phenomenon, and of great importance to the physician. The occurrence of albumen in this case is not constant, but alternating, it appears, before a trace of sugar can be observed, and when the sugar begins to form, it sometimes ceases again, and passing off it gives way to the albuminuria.

To demonstrate the presence of sugar in the urine is the principal means of satisfying oneself of the existence of diabetes mellitus. If the quantity of sugar is considerable, it is easily discovered in the alcoholic extract of the urine after evaporation; if only traces of sugar are present, the sulphate of copper is used to demonstrate its presence, as we shall see at another time.

Gallon pigment in the urine is constantly a sign of the liver being affected; we have already stated that this can be discovered by the addition of nitric acid; but to infer the presence of gall pigment from the colour of the urine is sometimes fallacious.

In some diseases fat is found in the urine; this partly shews itself to the naked eye as drops of fat, partly it is suspended in the urine in great quantity, so that the latter has a milky appearance; in this latter case albumen also is constantly present, and sometimes fibrine; here too the microscope is sufficient to convince ourselves of the presence of fat. The appearance of the chyle-urine, as Prout calls it, might lead one to suspect the presence of milk; but milk has been exceedingly seldom observed in the urine as a metastatical secretion separation; and it even seems that the majority of the cases where milk-urine has been spoken of, was only chylous urine; by dropping in dilute acetic acid, or hydrochloric acid, one might in an instant satisfy himself, whether casein or albumen, whether milk or albumen and fat imparted the white colour to the urine, it being known that casein is thrown down by milk and acetic acid. Among us the so-called chylous urine is a rarity: in southern countries, on the contrary, as the West Indies, it frequently makes its appearance. The urine is sometimes opalescent, sometimes milk-white; after it is voided, it is coagulated in a longer or shorter time, and presents a white tremulous mass resembling bile; fibrine is sometimes present only in very small quantity, or is altogether absent. Prout refers the seat of this disease to the organs of assimilation; however, regarding this point, we know but little that is positive. Drops of fat in the urine with albumen or only small traces of albumen occur in rapidly consuming diseases, as for instance, in phthisis; they are always an unfavourable sign. However, on the occurrence of fat in the urine persons should be particularly cautious against deception, as some fat might easily come into the vessel from without; sometimes there appears on the surface of the urine a film with ever varying colours, which may be taken for a fatty film, but which on a strict examination with the microscope, seems to be an amorphous matter, (commonly phosphate of lime) or consists of crystals (triple magnesian phosphate.)

The chemical and physical examination of mucus and pus is of great importance for establishing the diagnosis. The better and more satisfactory works on this subject have been published only in recent times; to these more especially belong those of Vogel, Hähule, Gruley, Jüterbock, Valentin, Woot, &c. The subject matter of them is to know, whether in the organ wherein the secretion of mucous is going on, suppuration is also taking place. The enquiries on this subject are among the most difficult; especially if it be taken into account, that mucous membranes when irritated pour out a secretion of a changeable quality, which in its appearance approximates very closely to pus. The varieties of pus and mucus lie not in the corpuscles, but in the fluid, dissolved parts; in the mucous liquid the mucous matter predominates, which is coagulated by water and acetic acid. Albumen is either not at all contained in it, or only in extremely small quantity; but if the mucus is secreted by constantly irritated mucous membranes, the albumen is augmented in the liquid, and the mucous matter is diminished. In the purulent liquid albumen and fat are predominant, and the mucous matter is only present in mere traces. From these marks the various habitudes of mucus and pus are derived. Mucus, as soon as it reaches the water, becomes invested with a covering of mucous matter; the same thing happens, when it is brought into dilute acetic acid, whereby it is immediately coagulated into a tolerably consistent jelly; if mucus be extended on a glass-plate, and then moistened with water, the instantaneous coagulation may then be observed with the naked eye, as well as with the microscope. This covering of coagulated mucous matter prevents the solution of the mucous balls in the water, and if air bubbles are contained therein, causes them to swim on the water. The pus is dissolved instantaneously in the water; its insoluble part sinks to the bottom, and forms there a stratum of a granular, purulent appearance; its soluble part is taken up by the water, and causes this to have a strong re-action in albumen. Those sputa in which globular, slippery mucous balls, not dissolved, are found lying indifferently at the bottom, or even floating, are always more favourable than those, where a uniformly dissolved, purulent-looking sediment has formed at the bottom of the vessel; the sputa are so much the more suspicious, the more the watery dissolved part allows us to discover the presence of albumen by boiling or nitric acid. The varieties in the habitude of mucus and purulent expectoration will be best seen by comparing the sputa of a person affected with pulmonary catarrh with those discharged in consequence of an opening of a vomica. With respect to the diagnosis of tubercular phthisis by the sputa, it is extremely difficult, and requires the greatest caution and attention; the mucous membrane of the respiratory organs is always placed in a state of irritation by the tubercular process, and in consequence of this a morbidly changed secretion is given off. The tubercles themselves either soften and suppurate, or may be expectorated while still in the crude state. In case the tubercles suppurate we always find with the rounded, tough and viscid expectorated mucus at the bottom of the vessel a purulent looking stratum, which, when observed with the microscope, consists of primary cells (mucus—or pus-corpuscles) and of a large mass of amorphous minutely divided matters. The watery part of the sputa always exhibits a greater or less quantity of albumen. It has been thought that, from the pieces of crude, tubercular masses, which are to be found in the expectoration of phthisical patients, we can determine the presence of tubercles in the lungs with great certainty; but the appearance of crude tubercular masses in the expectorated sputa is very rare, and no doubt in the majority of cases it was something of quite a different kind, more especially pieces of macerated bread, that was taken for tubercular masses, as both, when observed with the microscope as well as with the naked eye, bear some resemblance to each other. The crude tubercular mass appears in small, cheese-like grey pieces of a certain size, and present, when passed between glass-plates, a granular appearance, it becomes of a yellow colour when moistened with tincture of iodine. Morsels of macerated bread, which are sometimes found inclosed in balls of mucus, are similar in their habitude; under the microscope, together with the grumous amorphous matter they exhibit sometimes amylaceous granules contained within it; when moistened with tincture of iodine, the blue colour of iodine and starch shews itself. The detection and distinction of mucus and pus in the urine is in like manner extremely difficult; from the urine recently voided the pus sinks rather quickly and forms at the bottom of the vessel a purulent-looking mixed-coloured stratum tinged with blood; the urine standing over it constantly contains albumen. The mucus in the urine is slow in sinking; allows the urine to appear for a long time turbid or separates in clouds; the clear urinary fluid contains no albumen; only when the urine, which in this case easily happens, becomes ammoniacal, the mucus assumes a gelatinous viscid quality, and sinks rapidly to the bottom, still the pus in an ammoniacal urine becomes in like manner viscid. The chemical and microscopical examination of the pus itself affords some points for diagnosis; the pus from arthritic ulcers contains frequently uric acid or urate of soda, which is discovered either in the microscopical examination by the rhombic form of the uric acid crystals or by the pointed form of the crystals of the urate of soda, or by the purple red colour, which makes its appearance on heating the dried pus with nitric acid. The presence of uric acid in the pus warrants us in inferring with certainty the arthritic character of the ulcer. Normal pus has the consistence of a thick liniment; a yellowish colour, alkaline reaction, a not disagreeable, somewhat sweetish odour, and after a longer time it does not suffer its pus-corpuscles to fall. If the pus is thinnish or watery, it is not of good quality, being just as scrophulous pus. A dirty green or brown colour also indicates a bad quality of pus; to this belongs the reddish or chocolate-brown, even the darkish colour of the pus from the bones, and of the ichorous pus, of malignant pus, of carcinomatous or medullary fungoid ulcers, or of greenish syphilitic ulcers; to this latter a more or less strong acid re-action is peculiar; the odour in particular indicates the bad quality of the pus. In the malignant pus are found on a microscopical examination the pus-corpuscles partly irregular, knobby, partly destroyed, and floating as small amorphous particles or points. In the pus from the bones we sometimes find fragments of the destroyed substance of the bone, which may often be recognised by the naked eye.

The signs which may be obtained from the chemical examination of the urine for the establishing of the diagnosis and prognosis are, as it appears, of subordinate value. On account of the difficulty of collecting a large quantity of perspiration in diseases, our investigations are still very deficient. The mere assertions which we possess on the subject still require confirmation by repeated observations. The normal perspiration has constantly an acid reaction; but this free acid increases to an extraordinary degree in some diseases, as for instance, in rheumatism and gout. In these diseases it is found that the perspiration of the forehead, breast, and extremities intensely reddens litmus-paper in a rapid and extraordinary manner. According to Berend in putrid and typhus fever the perspiration not only should lose its acid, but even have an ammoniacal smell; according to Anselmino, in the critical sweat of a person labouring under rheumatic fever albumen should be present. Voigtel will have it that he has observed bloody sweat, and according to others this same thing is observed in putrid fever and typhus icterodes;—that the sweat of patients labouring under calculus, as also of gouty persons, contains uric acid and urate of soda, whereby, after the evaporation of the water, the body becomes covered over with a fine crystalline coating, has been frequently proved. In the sweat of icteric patients, as also of those labouring under the febris putrida biliosa, the colouring matter of the bile has been sometimes found. The red colouring matter, which imparts its colour to the lateritious sediment, has been found by Landerer in the sweat of the axillary glands of a fever patient. Blue sweat has been observed several times. In the colliquative sweats of hectic patients fat also has been found. All these signs are unsatisfactory; they do not appear constant in the various diseases, and for that reason possess far less importance than those derived from the blood or urine. The odour also of the perspiration is of particular importance. It is well known, that many medical men, on entering the sick-chamber, nay, even the sick-house, can infer the disease from the particular scent. The smell of the sweat can be the subject of chemical enquiry in but few cases; one may no doubt infer the presence of acetic acid in perspiration emitting an acid odour, and that of ammonia in sweat with an ammoniacal odour; but in most other cases the chemical examination is unattended with success; in like manner, the physician who possesses quickness of sense for distinguishing various odours, and who has exercised this sense by several years' experience, can be assisted through the sense of smell with tolerable certainty. The sweat of rheumatic and gouty persons has an acid odour; the sweat of putrid-fever patients and of scorbutic patients should have a putrid odour, that of syphilitic patients a sweetish one, that of itchy patients a mouldy smell, that of jaundic patients a musk-like smell, that of scrophulous patients the smell of sour dough, that of ague patients the smell of fresh-baked brown-bread.

The chemical and microscopical examination of the intestinal excretions is sometimes useful for the diagnosis. In disease, the fæcal matters deviate considerably from the normal state, as well in reference to their consistence as also to their mechanical and chemical composition. The deficiency of bile in the stools in cases of jaundice is sufficiently obvious. The great quantity of intestinal mucus floating in the watery dysenteric stools, which appears to the naked eye sometimes as a whey-like fluid with cheesy-flakes mixed through it, sometimes as a purulent mucus mixed with water, sometimes red-coloured with blood, and sometimes colourless, can be discovered with certainty only by means of the microscope; sometimes we find portions of coagulated exuded lymph, in which by proper management with the microscope we can recognize the fibrous structure of the coagulated fibrinous matter. The typhous stools thus readily dividing themselves into two strata exhibit to view by means of the microscope an extraordinary quantity of prismatic crystals of the triple-magnesian phosphate, besides mucus-corpuscles, and fragments of the epithelium of the intestinal mucous membrane connected together. In like manner, in intestinal typhus, the fluid stools divide themselves into two strata, of which the inferior, purulent-looking, is recognized under the microscope as constituted of mucus and pus-corpuscles; the superior stratum, in which these same corpuscles are still suspended, and sometimes fatty globules may also be discovered, is generally distinguished by a considerable quantity of dissolved albumen. The watery stools in cholera present under the microscope, among other objects, numerous crystals of triple magnesian phosphate; the purple red colour which these stools assume, when they are mixed with nitric acid, seems characteristic.

We may now notice the peculiar qualities of the vomiting which occurs in violent inflammations of the abdominal organs, and in carcinoma of the stomach; in the former case a watery, slightly mucous, green-coloured fluid, often extremely abounding in fat, is vomited forth; the fatty contents of which may be distinguished even with the naked eye, but still better with the microscope; in the second place, the mucous chocolate-coloured discharges contain a dark-coloured amorphous matter, several large yellow-coloured cells, which are filled with a granular mass, which also contains small fatty globules mixed with them. Besides a more or less considerable quantity of fatty drops seems to be peculiar to this vomiting.

